# Female nursing graduate students’ stress and health: the mediating effects of sense of coherence and social support

**DOI:** 10.1186/s12912-021-00562-x

**Published:** 2021-03-11

**Authors:** Yu Jin Jeong, Chin Kang Koh

**Affiliations:** 1 College of Nursing, Seoul National University, Seoul, Republic of Korea; 2 College of Nursing & The Research Institute of Nursing Science, Seoul National University, 103 Daehak-ro, Jongno-gu, Seoul, 03080 Republic of Korea

**Keywords:** Stress, Sense of coherence, Social support, Health status

## Abstract

**Background:**

Ninety-five percent of nursing graduate students in South Korea are women, and most are often engaged in both academic coursework and work outside of the academic environment. Nursing graduate students often experience stress leading to physical and mental health problems that negatively affect their academic performance and persistence during graduate programs. The purpose of this study was to test multiple mediation effects of sense of coherence (SOC) and social support in the relationship between stress and health status of nursing graduate students.

**Methods:**

The participants of this study were 231 female nursing graduate students from 14 universities. Data were collected using an online survey conducted between August and October 2019. Bootstrap techniques using the PROCESS macro for SPSS software were applied to assess the multi-mediating effects.

**Results:**

The total effect (B = − 12.29, *p* < .001) and direct effect (B = − 7.07, *p* < .001) of perceived stress on health status were significant. Perceived stress had negative direct effects on social support (B = − 0.41, *p* < .001) and SOC (B = − 5.77, *p* < .001). SOC had a positive direct effect on health status (B = 0.59, *p* < .001). However, social support was not a significant predictor of health status (B = 1.24, *p =* .232). In addition, there was a positive direct effect of social support on SOC (B = 5.23, *p* < .001). Furthermore, the indirect effect of perceived stress on health status through SOC was significant (B = − 3.42, 95% CI = − 5.2616, − 1.8906). There was also a significant indirect effect of perceived stress on health status through social support and SOC (B = − 1.28, 95% CI = − 2.1663, − 0.5992).

**Conclusion:**

It is necessary to create strategies that enhance nursing graduate students’ SOC and social support to reduce their perceived stress and to improve their health status.

## Background

Studying a graduate degree prepares nurses to play key roles in clinical leadership and scientific research to improve nursing care [1]. Therefore, graduate programs are essential for nursing as a profession and allow nursing students further academic development. However, there are various challenges for nursing graduate students, including a difficulty balancing several responsibilities related to their family, work, and school [2].

In previous studies of master’s and doctoral nursing students, nursing graduate students have been described as predominantly female [3, 4]. Female graduate students have been reported to have higher attrition rate than male students [5], and family issues such as child care responsibilities, marital problems, and death of a close family member, and lack of stable funding are major factors that constrain their academic progress [6]. Moreover, a study with Korean female graduate students revealed that physical and mental exhaustion due to their multiple roles lead to leave of absence from schools and delayed academic progress.

Moreover, nursing graduate students often work full-time while studying [3, 4]. Thus, they face challenges in balancing academic achievement and home and work management. As housework and parenting are traditionally considered the responsibilities of women, female graduate students strive to fulfill their roles for the family and often feel guilty when they are not able to perform their roles optimally [7]. Moreover, it is also reported that nursing graduate students experience difficulties in their workplace, such as lack of understanding from coworkers and schedule arrangements [8].

Nursing graduate students experience stress due to the aforementioned challenges, a stress that tends to increase as the program goes on [2, 7, 9–13]. For example, Maville et al. [10] found that nursing students perceived their stress levels to be moderate to high during a nurse practitioner program. Additionally, Cohen [14] reported that nursing doctoral students experienced stress related to doctoral persistence, and that female students experienced poor communication with others when seeking support and felt isolated and exhausted during their doctoral program. Moreover, nursing graduate students have difficulty with managing personal relationships, scheduling conflicts, and family management [15]. Further, nursing graduate students’ stress is related to physical and mental health problems, such as insomnia, fatigue, frequent gastrointestinal diseases, anxiety, and depression [7, 8, 16].

Antonovsky established sense of coherence (SOC) as a key concept of his salutogenic model. SOC is “a generalized orientation toward the world which perceives it, on a continuum, as comprehensible, manageable, and meaningful” [17], p.15. SOC enables individuals to cope effectively with stressors and plays a significant role in determining the individual’s location and movement on the health continuum [18]. Additionally, generalized resistance resources (another key concept of Antonovsky’s salutogenic model) are defined as “a property of a person, a collective or a situation which, as evidence or logic has indicated, facilitated successful coping with the inherent stressors of human existence” [17], p.15. Generalized resistance resources play a decisive role in determining movement toward the healthy end of the continuum [19]. Social support has been studied as a generalized resistance resource in the salutogenic model in previous studies [20, 21]. Previous research has shown that support from family, friends, colleagues, and faculty helps students cope with stress [7, 8, 11].

Studies dealing with graduate students’ health problems commonly state that graduate students experience high levels of stress (similar to vulnerable groups) that can be negatively affected by physical and mental health. Despite this, most studies dealing with the health problems of students in universities have targeted undergraduate students; only recently, studies have been conducted on graduate students including nursing majors [7, 8, 13, 16, 22, 23]. Thus, the purpose of this study was to identify the mediating effects of social support and SOC in the relationship between perceived stress and health status of female nursing graduate students, based on the salutogenic model.

## Methods

### Study design and participants

This cross-sectional study investigates the relationship among perceived stress, social support, SOC, and health status of female nursing graduate students. Based on the salutogenic model of Antonovsky, the multi-mediating effects of social support and SOC between stress and health status were tested. This study included 231 female graduate students studying master’s and doctoral programs in 14 graduate schools of nursing in South Korea.

### Procedure

The data were collected from August 2019 to October 2019, using online questionnaires via Survey Monkey (https://surveymonkey.com). Recruitment documents and links to online questionnaires were distributed through social network services through which nursing graduate students communicated and formed online network groups. All participants were informed about the study through recruitment documents. Nursing graduate students could choose to participate or not by clicking on the link to the online survey. To avoid replication, IP address of every respondent was checked when online questionnaires were received. There were 248 respondents in total, of which data from 231 were analyzed in this study to test the model.

### Measurements

#### Perceived stress

Nursing graduate students’ perceived stress was measured with a seven-item instrument developed specifically for this study based on previous studies focusing on graduate students’ stress experience [2, 3, 7, 10–13, 22]. The seven items assessed stressors commonly reported by graduate students in field-relevant literature, including “class requirements (e.g., presentations, examinations, and assignments),” “research/thesis writing,” “relationship with professors,” “career uncertainty after obtaining degree,” “scheduling conflicts/time shortage,” “financial problems,” and “balancing family, social, work, and academic responsibilities.” Participants were tasked to read each item and answer how often they experienced stress due to each aspect during their graduate program; all items were scored on a 5-point Likert scale (0 = “never,” 1 = “rarely,” 2 = “sometimes,” 3 = “often,” 4 = “always”). Higher mean scores indicated a higher level of perceived stress. In this study, Cronbach’s alpha for this scale was 0.70, and the factor loadings as a validity test of the items ranged from 0.43 to 0.65 in confirmatory factor analysis.

#### Social support

Social support was assessed using the Multidimensional Scale of Perceived Social Support (MSPSS). This scale was created by Zimet et al. [24], and was translated into Korean by Shin and Lee [25]. This instrument consists of 12 items, each scored on a Likert scale ranging from 1 (“very strongly disagree”) to 7 (“very strongly agree”). It includes three subscales: significant other, family, and friends. Higher mean scores indicate a higher level of social support. In this study, Cronbach’s alpha for the whole scale was 0.94, while it ranged from 0.91 to 0.92 for the subscales (significant other = 0.91; family = 0.91; friends = 0.92).

#### Sense of coherence

Nursing graduate students’ SOC was assessed using Antonovsky’s 13-item version of SOC (SOC-13) [26]. This instrument consists of 13 items on a 7-point Likert scale. SOC-13 consists of three subscales: comprehensibility (5 items), manageability (4 items), and meaningfulness (4 items). The total score is the sum of the 13 items and ranges from 13 to 91. A high total score indicates a strong SOC. We obtained permission to use the Korean version of the SOC-13 scale through the Society for Theory and Research on Salutogenesis (STARS). In this study, Cronbach’s alpha for the total scale was 0.84 and subscale values were 0.63 to 0.66 (comprehensibility = 0.66; manageability = 0.63; meaningfulness = 0.64).

#### Health status

The 36-Item Health Survey (version 1.0) developed by RAND Corporation was used to assess nursing graduate students’ health status [27]. This scale was translated into Korean by Koh et al. [28]. The 36-Item Health Survey evaluates eight health concepts: physical functioning, bodily pain, role limitations due to physical health problems, role limitations due to personal or emotional problems, emotional well-being, social functioning, energy/fatigue, and general health perceptions. Each question was transformed into a scale ranging from 0 to 100 according to the RAND scoring method, and the average scores of eight subscales were calculated. The scores for the eight subscales ranged from 0 to 100, with a higher score indicating better health status. In this study, the total mean score of the eight subscales was used to assess health status. In this study, Cronbach’s alpha for this scale ranged from 0.62 to 0.88, and factor loadings of the eight subscales ranged from 0.38 to 0.78 in confirmatory factor analysis.

#### Participants’ characteristics

This questionnaire included seven items on sociodemographic characteristics. These items included age, marital status, enrollment program, enrollment status, employment status, and clinical experience.

### Data analysis

Data were analyzed using AMOS 23, SPSS 22, and SPSS PROCESS macro version 3.5 software. Nursing graduate students’ characteristics were presented using descriptive statistics, including frequencies, percentages, means, and standard deviations (SDs). Participants’ perceived stress, social support, SOC, and health status were expressed as means ± SD. To examine the normality of the study variables, skewness and kurtosis were computed. Bivariate correlations were used to examine the associations among participants’ characteristics, perceived stress, social support, SOC, and health status. Moreover, confirmatory factor analysis was carried out using AMOS 23.0 software to test the validity of the scales.

Model 6 of the SPSS PROCESS macro [29] was used to test the multiple mediation model. In the analysis, perceived stress was entered as an independent variable and health status as the dependent variable. In addition, social support and SOC were entered simultaneously as mediating variables. Age, marital status, and enrollment program were treated as covariates in the analysis. This procedure was based on 5000 bootstrap samples and a bias-corrected 95% confidence interval.

## Results

### Participants’ characteristics

Participants’ characteristics are summarized in Table 1. A total of 231 female nursing graduate students participated in this study. The mean age of participants was 32.87 years (SD = 5.56). Of all participants, 56.3% were single. Most participants (68.0%) were enrolled in a master’s program, and 72.7% were part-time students. The majority (70.6%) of participants were employed full-time, and their mean clinical experience was 8.22 years (SD = 4.94). The mean perceived stress score was 2.21 (SD = 0.58). In terms of social support and SOC, the mean scores were 5.58 (SD = 0.88) and 57.05 (SD = 11.09), respectively. Overall, participants had a mean health status score of 65.65 (SD = 15.09). All the variables were normally distributed, with skewness and kurtosis ranging from − 2 to 2.

**Table 1:** Participants’ Characteristics (*N* = 231)

Variables	n (%) / mean ± SD
Age (in years)	32.81 ± 5.56
Marital status	
Single	130 (56.3)
Married	101 (43.7)
Enrollment program	
Master’s program	157 (68.0)
Doctoral course	74 (32.0)
Enrollment status	
Full-time	63 (27.3)
Part-time	168 (72.7)
Employment status	
Unemployed or part-time	68 (29.4)
Full-time	163 (70.6)
Clinical experience (in years)	8.22 ± 4.94
Perceived stress	2.21 ± 0.58
Social support	5.58 ± 0.88
Sense of coherence	57.05 ± 11.09
Health status	65.65 ± 15.09

Abbreviations: SD, standard deviation

### Correlations among participants’ characteristics, perceived stress, social support, sense of coherence, and health status

Table 2 presents Pearson’s correlation coefficients of the association between study variables. Of participants’ characteristics, age had positive correlation with perceived stress (r = .139, *p* = .034) and SOC (r = .133, *p* = .043). In addition, marital status was positively associated with perceived stress (r = .154, *p* = .019), social support (r = .163, *p* = .013), and SOC (r = .144, *p* = .029). Enrollment program was positively associated with perceived stress (r = .284, *p* < .001) but negatively associated with health status (r = −.158 *p* = .016).

**Table 2:** Correlations between study variables (*N* = 231)

	1	2	3	4	5	6	7	8	9	10
**1. Age (in years)**	1									
**2. Marital status**^a^	.425^***^	1								
**3. Enrollment program**^b^	.317^***^	.255^***^	1							
**4. Enrollment status**	−.158^*^	−.009	−.121	1						
**5. Employment status**	−.070	−.158^*^	−.371^***^	.266^***^	1					
**6. Clinical experience (in years)**	.703^***^	.280^***^	−.029	−.050	.141^*^	1				
**7. Perceived stress**	.139^*^	.154^*^	.284^***^	−.093	−.083	.028	1			
**8. Social support**	−.030	.163^*^	.062	.053	.035	−.066	−.224^**^	1		
**9. Sense of coherence**	.133^*^	.144^*^	.035	.013	.015	.085	−.358^***^	.486^***^	1	
**10. Health status**	−.103	−.047	−.158^*^	.039	−.026	−.101	−.480^***^	.340^***^	.550^***^	1

^a^Marital status (0 = single, 1 = married); ^b^Enrollment program (0 = master’s program, 1 = doctoral course); ^*^p < .05; ^**^p < .01; ^***^p < .001

Moreover, results showed that health status was negatively correlated with perceived stress (r = −.480, *p* < .001) but positively correlated with SOC (r = .550, *p* < .001) and social support (r = .340, *p* < .001). SOC had a significant and negative correlation with perceived stress (r = −.358, *p* < .001), whereas it had a positive correlation with social support (r = .486, *p* < .001). Lastly, social support was negatively associated with perceived stress (r = −.224, *p* = .001).

### Multiple mediation analyses

**Fig. 1:**
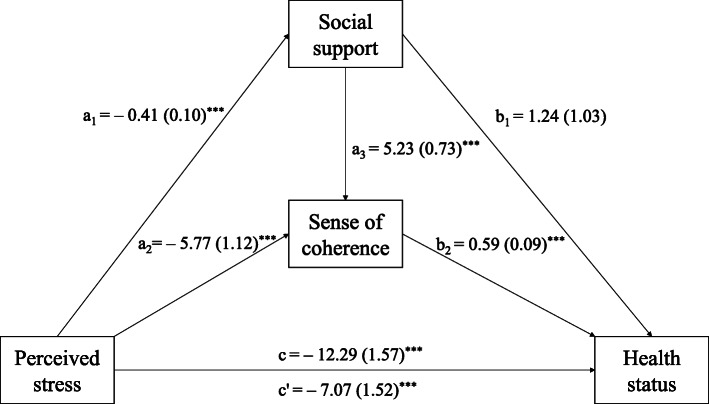
Results of the mediation analysis, controlling for age, marital status, and enrollment program (*N* = 231). The parameter estimates were presented as B (SE). a_1_ = direct effect of perceived stress on social support; a_2_ = direct effect of perceived stress on sense of coherence; a_3_ = direct effect of social support on sense of coherence; b_1_ = direct effect of social support on health status; b_2_ = direct effect of sense of coherence on health status; c’ = direct effect of perceived stress on health status; c = total effect of perceived stress on health status. ^*^p < 0.05, ^**^p < 0.01, ^***^p < 0.001

The results of the multiple mediation model are presented in Fig. 1 and Table 3. The results revealed that perceived stress had a significant effect on health status (path c: B = − 12.29, SE = 1.57, *p* < .001; R^2^ = 0.23). Perceived stress had negative direct effects on social support (Path a_1_: B = − 0.41, SE = 0.10, *p* < .001) and SOC (path a_2_: B = − 5.77, SE = 1.12, *p* < .001). In addition, among the mediating variables, social support had a positive direct effect on SOC, another mediating variable (path a_3_: B = 5.23, SE = 0.73, *p* < .001). Moreover, SOC (path b_2_: B = 0.59, SE = 0.09, *p* < .001) had significant direct effects on health status, whereas perceived stress (path c’: B = − 7.07, SE = 1.52, *p* < .001) had a negative direct effect on health status. However, social support (path b_1_: B = 1.24, SE = 1.03, *p =* .232) was not a significant predictor of health status (Fig. 1).

**Table 3:** Indirect effects of perceived stress on health status mediated by social support and sense of coherence (*N* = 231)

Effect		B	SE	95% CI	
				LLCI	ULCI
Total indirect effect of perceived stress on health status		−5.21	1.06	−7.4150	−3.3087
Indirect effect 1	Perceived stress → social support → health status	−0.51	0.54	− 1.6872	0.4808
Indirect effect 2	Perceived stress → sense of coherence → health status	−3.42	0.86	−5.2616	−1.8906
Indirect effect 3	Perceived stress → social support → sense of coherence → health status	−1.28	0.40	−2.1663	−0.5992

Bootstrap sample size = 5000; *CI* confidence interval, *SE* standard error, *LLCI* lower limit confidence interval, *ULCI* upper limit confidence interval

The total indirect effect of perceived stress on health status was found to be significant (B = − 5.21, SE = 1.06, 95% CI = − 7.4150, − 3.3087). The indirect effect of perceived stress on health status through SOC was significant (a_2_b_2_: B = − 3.42, SE = 0.86, 95% CI = − 5.2616, − 1.8906). Furthermore, there was a significant indirect effect of perceived stress on health status through the two mediators (social support and SOC) in serial (a_1_a_3_b_2_: B = − 1.28, SE = 0.40, 95% CI = − 2.1663, − 0.5992). However, the indirect effect of perceived stress on health status through social support was not significant (a_1_b_1_: B = − 0.51, SE = 0.54, 95% CI = − 1.6872, 0.4808).

### Discussion

This study identified how SOC, a key factor in Antonovsky’s salutogenic model, affects the relationship between stress and health status of female nursing graduate students. It was explored whether social support as a generalized resistance resource contributed to improving SOC.

In this study, SOC had a negative correlation with perceived stress, whereas it had a positive correlation with health status. In addition, perceived stress had an indirect effect on health status through SOC. These results indicate that high levels of SOC can reduce the effect of perceived stress on health status. The results of this study are consistent with those of Kenne Sarenmalm et al., who reported that SOC buffers the negative effect of stressors on health status [30], and with the results of Wilczek-Rużyczka et al., who reported that SOC acts as an important resource, buffering the negative effect of mental load on the well-being of nurses [31].

According to the salutogenic model, SOC is a decisive factor affecting health outcomes and well-being [32]. Previous studies have found that SOC is related to perceived health and other aspects of well-being [33–35]. People with strong SOC employ appropriate strategies to cope successfully with stressors [30], and they are more likely to utilize various physical and human resources around them to cope with stressful stimuli [36]. Conversely, people with weak SOC may be unable to initiate coping behaviors when faced with difficulties or may have difficulty dealing with stress effectively due to lack of confidence in coping [37]. Depending on the degree of SOC, the way individuals cope with stressful changes and the level of psychological distress and physiological responses they experience may differ [37].

Furthermore, in the multi-mediating model used in this study, social support (a generalized resistance resource) had an indirect effect on health status through SOC, although its direct effect was not significant. This supports Antonovsky’s findings, which suggest that generalized resistance resources may facilitate effective responses to stressors and contribute to the development of individuals’ SOC [18, 19]. Moreover, the results of this study are consistent with other studies. For example, Kase et al. [20] reported that social support was related to Japanese university students’ mental health status through SOC.

The current study showed that nursing graduate students with higher levels of social support were more likely to have stronger SOC. Consequently, high levels of social support can reduce the effects of stress on nursing graduate students’ health status by enhancing SOC. In previous studies, nursing graduate students reported family, friends, and colleagues to be major support resources [7, 11]. The importance of support from faculty has also been recognized during their degree program [14]. However, some may feel more isolated during their graduate program because of failure to communicate with people from whom they expected to receive support [14]. Thus, appropriate support needs to be provided to nursing graduate students.

Negative health outcomes due to stress in graduate programs have been reported. Some serious diseases, such as cancer, may occur among graduate students experiencing extreme stress [14]. To prevent decline in health due to stress among nursing female graduate students, strategies at the individual, family, and university levels need to develop strategies to decrease stressors and create a strong social support system. In particular, there are various types of academic programs that may provide opportunities for grants, scholarships, and funding for students with financial difficulties; facilitate course work and thesis with technical support for writing, computer, or statistics skills; supportive relationships with professors; and develop counseling services to help manage role strain and time conflict.

In 2019, the number of nursing graduate students in South Korea was 3925 [38], more than three times that of 1999 (1264) [39]. Most nursing graduate students (95%) are women [38]. Despite the increase in women’s advancements in the academic area, the institutional and legal support for female graduate students is still insufficient in Korea [40]. Responsibilities related to the family are primarily imposed on women, which can increase the stress of female nursing students who play various roles at school, work, and home. Therefore, social and institutional support systems should be established to help them pursue the degree course. It is especially necessary to establish legal grounds for supporting childbirth and childcare among female students [40]. Meanwhile, as more than half of the participants in our study were single, support from the workplace is expected to have a particularly important impact. In the clinical field, an organizational culture should be created to encourage graduate school entrance, and practical support such as substitute workers should be provided [8].

The main strength of this study is that it deepens our understanding of stress and health of female nursing graduate students, who have been relatively overlooked in prior literature. However, this study has some limitations. First, this study was conducted in the graduate schools that the researchers could access; thus, the results of this study should be generalized with caution. In addition, this study tested only social support among various generalized resistance resources that were present in the salutogenic model. Therefore, further studies are needed to identify the effects of other resistance resources.

## Conclusions

This study was conducted to test the mediating effects of SOC and social support on the relationship between perceived stress and health status of female nursing graduate students, based on Antonovsky’s salutogenic model. The findings of this study indicate that SOC is an important factor in reducing stress and improving the health status of nursing graduate students. In addition, this study demonstrated that social support as a generalized resistance resource affects health status by strengthening SOC. Thus, to improve nursing graduate students’ health status and to support their academic success, it is necessary to create and implement intervention programs that enhance SOC and social support.

## Data Availability

The dataset supporting the conclusions is available from the corresponding author on reasonable request.

## Abbreviations

SOC: Sense of coherence
SD: Standard deviation
SE: Standard error
CI: Confidence interval

## References

[1] Institute of Medicine (2011). The Future of nursing: leading change, advancing health.

[2] Baldwin S (2013). Exploring the experiences of nurses studying professional doctorates. Br J Nurs.

[3] Brown K, Anderson-Johnson P, McPherson AN (2016). Academic-related stress among graduate students in nursing in a Jamaican school of nursing. Nurse Educ Pract.

[4] Reilly JER, Fitzpatrick JJ (2009). Perceived stress and sense of belonging doctor of nursing practice students. J Prof Nurs.

[5] Ferreira M (2003). Gender issues related to graduate student attrition in two science departments. Int J Sci Educ.

[6] Maher MA, Ford ME, Thompson CM (2004). Degree progress of women doctoral students: factors that constrain, facilitate, and differentiate. Rev High Ed.

[7] Lee KS, Park EJ, Kim HJ, Ahn HR (2011). An exploration on the stress of Korean graduate nursing students: using of focus group research method. J Korean Acad Psychiatr Ment Health Nurs.

[8] Lim Y, Lee S, Song H, Park H (2018). The meaning of study in the convergence role of married nursing graduate students: focusing on doctoral students. J Korea Converg Soc.

[9] Ratanasiripong P, Park JF, Ratanasiripong N, Kathalae D (2015). Stress and anxiety management in nursing students: biofeedback and mindfulness meditation. J Nurs Educ.

[10] Maville JA, Kranz PL, Tucker BA (2004). Perceived stress reported by nurse practitioner students. J Am Acad Nurse Pract.

[11] Kang MJ, Kim YH, Cho YS, Lee SR, Kim KR, Kim YS (2003). A phenomenological study on the experience of female doctoral candidates in nursing. Qual Res.

[12] Volkert D, Candela L, Bernacki M (2018). Student motivation, stressors, and intent to leave nursing doctoral study: a national study using path analysis. Nurse Educ Today.

[13] Shin JH, Kang KO, Lee SJ, Kim HS (2016). Graduate School experiences of married women in the nursing profession. J Digit Converg.

[14] Cohen SM (2011). Doctoral persistence and doctoral program completion among nurses. Nurs Forum.

[15] Kenty JR (2000). Stress management strategies for women doctoral students. Nurse Educ.

[16] Nogueira-Martins LA, Fagnani Neto R, Macedo PCM, Cítero VA, Mari JJ (2004). The mental health of graduate students at the Federal University of São Paulo: a preliminary report. Braz J Med Biol Res.

[17] Antonovsky A (1996). The salutogenic model as a theory to guide health promotion. Health Promot Int.

[18] Antonovsky A (1993). The structure and properties of the sense of coherence scale. Soc Sci Med.

[19] Antonovsky A (1979). Health, stress, and coping.

[20] Kase T, Endo S, Oishi K (2016). Process linking social support to mental health through a sense of coherence in Japanese university students. Ment Health Prev.

[21] Tsuno YS, Yamazaki Y (2012). Relationships among sense of coherence, resources, and mental health in urban and rural residents in Japan. BMC Public Health.

[22] Kernan W, Bogart J, Wheat ME (2011). Health-related barriers to learning among graduate students. Health Educ.

[23] Kim EH, Lim YO, Park GS, Kim NY (2008). A grounded theory approach on the multiple role experience of married women graduate students. Korean J Adult Nurs.

[24] Zimet GD, Dahlem NW, Zimet SG, Farley GK (1988). The multidimensional scale of perceived social support. J Pers Assess.

[25] Shin JS, Lee YB (1999). The effects of social supports on psychosocial well-being of the unemployed. Korean J Soc Welf.

[26] Antonovsky A (1987). Unraveling the mystery of health: how people manage stress and stay well.

[27] Ware JE, Sherbourne CD (1992). The MOS 36-item short-form health survey (SF-36). I. Conceptual framework and item selection. Med Care.

[28] Koh SB, Chang SJ, Kang MG, Cha BS, Park JK (1997). Reliability and validity on measurement instrument for health status assessment in occupational workers. J Prev Med Public Health.

[29] Hayes AF (2017). Introduction to mediation, moderation, and conditional process analysis: a regression-based approach.

[30] Kenne Sarenmalm EK, Browall M, Persson LO, Fall-Dickson J, Gaston-Johansson F (2013). Relationship of sense of coherence to stressful events, coping strategies, health status, and quality of life in women with breast cancer. Psychooncology..

[31] Wilczek-Rużyczka E, Dębska G, Pasek M, Zwierzchowska M (2019). The mediational effect of coherence on the relationship between mental load and job burnout among oncology nurses. Int J Nurs Pract.

[32] Sagy S, Antonovsky A (1992). The family sense of coherence and the retirement transition. J Marriage Fam.

[33] Rai A, Sindhu A, Dudeja P, Sirohi YS, Mukherji S (2018). Sense of coherence and self reported health amongst medical students: a cross sectional study. Med J Armed Forces India.

[34] Kuwato M, Hirano Y (2020). Sense of coherence, occupational stressors, and mental health among Japanese high school teachers in Nagasaki prefecture: a multiple regression analysis. BMC Public Health.

[35] Malagon-Aguilera MC, Suñer-Soler R, Bonmatí-Tomas A, Bosch-Farré C, Gelabert-Vilella S, Juvinyà-Canal D (2019). Relationship between sense of coherence, health and work engagement among nurses. J Nurs Manag.

[36] Mato M, Tsukasaki K (2019). Factors promoting sense of coherence among university students in urban areas of Japan: individual-level social capital, self-efficacy, and mental health. Glob Health Promot.

[37] McSherry WC, Holm JE (1994). Sense of coherence: its effects on psychological and physiological processes prior to, during, and after a stressful situation. J Clin Psychol.

[38] Ministry of Education. Education statistical year book; 2019. https://kess.kedi.re.kr/index. Accessed 24 Mar 2020 (in Korean).

[39] Ministry of Education. Education statistical year book; 2000. https://kess.kedi.re.kr/index. Accessed 9 Dec 2020 (in Korean).

[40] Seo J (2015). The phenomenology of the graduate student mom’s study-childrearing experiences in the research universities in South Korea. Womens Stud.

